# RT-QuIC detection of chronic wasting disease prion in platelet samples of white-tailed deer

**DOI:** 10.1186/s12917-024-04005-y

**Published:** 2024-04-23

**Authors:** Estela Kobashigawa, Sherri Russell, Michael Z. Zhang, Emily A. Sinnott, Michael Connolly, Shuping Zhang

**Affiliations:** 1 Veterinary Medical Diagnostic Laboratory, College of Veterinary Medicine, University of Missouri, 901 E. Campus Loop, Columbia, MO USA; 2 Department of Veterinary Pathobiology, College of Veterinary Medicine, University of Missouri, 901 E. Campus Loop, Columbia, MO USA; 3 Missouri Department of Conservation, 2901 W Truman Blvd, Jefferson City, MO USA; 4 The Molecular Foundry, Lawrence Berkeley National Laboratory, 67 Cyclotron Rd, Berkeley, CA USA

**Keywords:** CWD, Deer, Platelet, RT-QuIC, RAF, Sensitivity, Specificity, Probability

## Abstract

**Background:**

Chronic wasting disease (CWD) is a prion disease of captive and free-ranging cervids. Currently, a definitive diagnosis of CWD relies on immunohistochemistry detection of PrP^Sc^ in the obex and retropharyngeal lymph node (RPLN) of the affected cervids. For high-throughput screening of CWD in wild cervids, RPLN samples are tested by ELISA followed by IHC confirmation of positive results. Recently, real-time quacking-induced conversion (RT-QuIC) has been used to detect CWD positivity in various types of samples. To develop a blood RT-QuIC assay suitable for CWD diagnosis, this study evaluated the assay sensitivity and specificity with and without ASR1-based preanalytical enrichment and NaI as the main ionic component in assay buffer.

**Results:**

A total of 23 platelet samples derived from CWD-positive deer (ELISA + /IHC +) and 30 platelet samples from CWD-negative (ELISA-) deer were tested. The diagnostic sensitivity was 43.48% (NaCl), 65.22% (NaI), 60.87% (NaCl-ASR1) or 82.61% (NaI-ASR1). The diagnostic specificity was 96.67% (NaCl), 100% (NaI), 100% (NaCl-ASR1), or 96.67% (NaI-ASR1). The probability of detecting CWD prion in platelet samples derived from CWD-positive deer was 0.924 (95% CRI: 0.714, 0.989) under NaI-ASR1 experimental condition and 0.530 (95% CRI: 0.156, 0.890) under NaCl alone condition. The rate of amyloid formation (RFA) was greatest under the NaI-ASR1 condition at 10^–2^ (0.01491, 95% CRI: 0.00675, 0.03384) and 10^–3^ (0.00629, 95% CRI: 0.00283, 0.01410) sample dilution levels.

**Conclusions:**

Incorporation of ASR1-based preanalytical enrichment and NaI as the main ionic component significantly improved the sensitivity of CWD RT-QuIC on deer platelet samples. Blood test by the improved RT-QuIC assay may be used for antemortem and postmortem diagnosis of CWD.

**Supplementary Information:**

The online version contains supplementary material available at 10.1186/s12917-024-04005-y.

## Background

Chronic wasting disease (CWD) is a prion disease of captive and free-ranging cervids, including mule deer (*Odocoileus hemionus*), white-tailed deer (*Odocoileus virginianus*), elk (*Cervus canadensis*), sika deer (*Cervus nippon*), and moose (*Alces alces*) [1, 2]. Clinical manifestations of CWD include weight loss, progressive weakness, tremors, nervousness, and death [3]. The mechanism of the disease involves the conversion (or misfolding) of a native alpha-helix rich, protease-sensitive protein PrP^C^ (C stands for cellular) to a beta-structure-rich, protease-resistant, self-replicating conformation, denoted PRP^Sc^ (Sc stands for scrappies) [3]. Like other prion diseases, the neuropathology of CWD is characterized by spongiform degeneration, neuronal loss, glial activation and the accumulation of PrP^Sc^ [1–3]. Although PrP^Sc^ is mostly present in the brain and lymph nodes of affected animals, lower levels have been detected in blood and body fluids, such as saliva, urine, and the environment [4, 5]. The detection of CWD PrP^Sc^ in body fluids suggests that the disease can be spread via direct contact between cervids and contact with soil, food, and water that have been contaminated. It has also been shown that CWD blood infectivity is associated with platelets and B cells [6] and the potential for transmission increases when animals gather in larger, concentrated numbers ([7–9]. According to the United States Centers for Disease Control and Prevention, there is no strong evidence for the occurrence of CWD in people, and it is not known if people can be infected with CWD prions (https://www.cdc.gov/prions/cwd/index.html). However, it is important to keep the agents of all known prion diseases from entering the human food chain (https://www.cdc.gov/prions/cwd/index.html).

Diagnosis of CWD can be achieved using several technologies, such as immunohistochemistry (IHC), Western Blot (WB), and Enzyme Linked Immunosorbent Assay (ELISA) [10]. Evaluation of the dorsal motor nucleus of the vagus in the obex region of the brainstem and the medial retropharyngeal lymph nodes (RPLN) by IHC is considered the gold standard and required by the United States Department of Agriculture (USDA) for the Voluntary CWD Certification Program. Western Blot is specific and may be used to differentiate strains but is time-consuming and not suitable for high throughput testing. ELISA is utilized routinely by veterinary laboratories to screen for CWD prion in wild and free-range cervids and ELISA positive results are confirmed by IHC. Over the last two decades, emergence of prion amplification or conversion has revolutionized the field of prion diagnostics and research. The protein misfolding cyclic amplification (PMCA) takes advantage of the ability of PrP^Sc^ to induce a conformational change in Prp^C^ and the amplified PrP^Sc^ is detected by a second method, such as WB [11–13]. Similarly, real-time quaking-induced conversion (RT-QuIC) assay relies on the use of recombinant PrP^C^ as a substrate and the prions in the samples under investigation as seeds to induce the conversion of PrP^C^ to PrP^Sc^ [14, 15]. The formation of Prp^Sc^ aggregates during RT-QuIC reaction is monitored in real time through the incorporation of an amyloid-sensitive dye into the aggregates. The advantages of RT-QuIC include suitability for high throughput testing and free of generating infectious prions. To understand CWD pathogenesis and identify suitable diagnostic specimens, RT-QuIC has been used to detect CWD prion in lymph nodes [16], urine [17], blood [18, 19], nasal brushing and recto-anal associated lymph tissue [20, 21], and feces [21, 22]. When compared to IHC-based diagnosis of CWD, the sensitivity of RT-QuIC detection of CWD prion in different samples varies widely, ranging from 100% on RPLN [16], 79% to 90% on blood [18, 19], 77.3% to 92% on RAMALT [20, 21], 60% for feces [21] to 34% on nasal brushing [20].

The sensitivity of prion detection can be improved by incorporating a pre-analytical enrichment procedure, such as sodium phosphotungstic acid (NaPTA) precipitation, antibody-tagging, or magnetic particle binding [18, 23, 24]. NaPTA precipitation involves incubating samples with sarkosyl and NaPTA with constant agitation followed by centrifugation to pellet the prion protein [18]. Antibody-tagging is based on the binding of monoclinal antibody specific to PrP^Sc^ conformation to detect prions [23]. The magnetic particle binding method utilizes superparamagnetic iron oxide beads to capture amplification-competent prions in samples [24]. In addition, a novel reagent, namely aggregated specific reagent 1 (ASR1), has been developed based on two prion protein-derived peptides (PrP19–30 and PrP100–111) [25–27]. ASR1 can selectively capture diverse misfolded proteins by interacting with a common supramolecular feature of protein aggregates [26]. Pre-treatment of samples with ASR1 enhanced the detection of prions in human plasma samples [27]. Besides preanalytical enrichment, Hofmeister anions from weakly hydrating to strongly hydrating could affect prion seeding activity via modifying the ionic environment of RT-QuIC [28, 29]. Sodium iodide, a weakly hydrated and solubilizing salt, improved the kinetic separation of prion-seeding in scrapie prion RT-QuIC assays, thereby affecting assay sensitivity and specificity [29].

To evaluate RT-QuIC using blood as CWD diagnostic sample, the present study compared the sensitivity and specificity of RT-QuIC on blood collected from CWD-positive and CWD-negative white-tailed deer harvested by hunters under different assay conditions, including NaCl as the main ionic component with and without ASR1-based preanalytical enrichment and NaI as the main ionic component with and without preanalytical enrichment.

## Materials and methods

### Diagnostic samples

As part of the Missouri Targeted Removal program, retropharyngeal lymph nodes (RPLN) and postmortem cardiac blood were collected from the white-tailed deer by the field agents of the Missouri Department of Conservation (MDC) immediately after the death of the animals. The protocols used were reviewed by the leadership committee of the Science Branch of MDC. Approximately 45 ml of postmortem cardiac blood were collected into a syringe containing 5 ml sodium citrate anticoagulant (Thermo Fisher Scientific) and mixed gently by slowly inverting the syringe at least 10 times. The blood samples were transported at room temperature to the VMDL for testing. Retropharyngeal lymph nodes (RPLN) and blood samples were submitted to the Veterinary Medical Diagnostic Laboratory (VMDL) for CWD diagnosis. This research utilized diagnostic samples to evaluate RT-QuIC assay under various conditions and did not involve the use of live animals. The research protocol was approved by the University of Missouri Institutional Biosafety Committee (protocol number: 11721 2.2).

### Determination of CWD status

RPLN samples were processed and tested in VMDL Diagnostic Serology section using ELISA according to the manufacturer’s instruction (HerdChek CWD Ag Test kit, IDEXX Laboratories, Westbrook, ME, USA). ELISA positive results were verified by immunohistochemistry (IHC) according to the protocol provided by National Veterinary Service Laboratory (NVSL-SOP-0878). ELISA and IHC were conducted by the VMDL Serology and Immunohistochemistry sections, respectively. When both RPLN samples from the same deer were tested positive by ELISA and confirmed by IHC, the deer was considered positive for CWD. In contrast, if both RPLN samples from the same deer were tested negative by ELISA, the deer was considered negative for CWD without IHC confirmation of ELISA result. The diagnostic results were blinded from the research team conducting RT-QuIC analysis.

### Preparation of platelet samples

A total of 23 platelet samples from CWD-positive deer and 30 platelet samples derived from CWD-negative white-tailed deer were included in this study. Upon arrival, blood samples were transferred from the collection syringes into 50 mL conical tubes (Thermo Fisher Scientific, Waltham, MA, USA). Platelets were isolated as previously described [30]. In brief, the tubes containing blood samples were subjected to a low-speed centrifugation (1,000 g) for 9 min at room temperature. The upper fraction (approximately 10 mL) of each tube consisting of platelet-rich plasma was transferred to a new 15 mL conical tube without anticoagulant (Thermo Fisher, Waltham, MA, USA). The tubes were centrifuged again at a high speed (3,000 g) for 20 min at room temperature. After the second centrifugation, supernatant containing platelet-poor plasma was removed and platelet pellet along with small volume of plasma (1 mL in total) was collected and stored at -80℃ prior to analysis [30].

### Preparation of ASR1 reagent

The ASR1 peptoid was synthesized on Rink amide resin according to previously reported automated solid phase synthesis techniques [31, 32]. This efficient and robust synthesis is achieved by a two-step monomer addition cycle, without using any main chain protecting groups. The first step is an acylation reaction of a resin-bound amine with bromoacetic acid, and the second step is a displacement reaction with a primary amine submonomer, to sequentially incorporate chemically distinct monomers along the growing peptoid chain with each iterative cycle [33]. The completed peptoid was cleaved from the resin using 95:2.5:2.5 trifluoroacetic acid:water:triisopropylsilane and purified by reverse phase HPLC. The ASR1 beads were generated by chemical conjugation of a thiolated ASR1 peptoid derivative to magnetic beads (Dynabeads™ M-270 Carboxylic Acid, Invitrogen, Carlsbad, CA, USA) as described previously [26]. The ASR1 reagent was prepared as a 30 mg/ml bead suspension in bead storage buffer (1 × PBS with 0.1% Sodium Azide, 0.01% Triton X-100). The ASR1 peptoid loaded on the beads was measured by quantitative ninhydrin assay to be 9.39 nmol/mg of beads. For preanalytical enrichment, 100 µL of platelet suspension was mixed with 30 µL of ASR1 reagent and incubated at room temperature for 1 h. After incubation, prion bound ASR1 beads were harvested using a magnetic separation rack and resuspended in 10 µL of PBS containing 0.1% SDS for RT-QuIC analysis.

### Performance of RT-QuIC

The platelet samples were subjected to three freeze–thaw cycles and serially diluted (10^–1^, 10^–2^, and 10^–3^) in PBS with 0.1% SDS. The diluted samples were tested by RT-QuIC. The assay master mix contained 20 mM Na2HPO4, 320 mM NaCl (or NaI), 1 mM EDTA, 50 µM Thioflavin T (Sigma-Aldrich, Burlington, MA, USA), and 0.1 mg/ml rPrP substrate (Syrian Hamster rPrP: 90–231, CWD Evolution). To each well of a 96-well black clear bottom plate (Greiner Bio-One, Monroe, NC, USA), 95 µl of the master mix was added followed by addition of 3 µL of the sample to be tested. Each run included 2 wells of negative control and 2 wells of positive control. The plate was sealed with Nunc Amplification Tape (Nalge Nunc International, Rochester, NY, USA) and incubated at 42℃ in a BMG FLUOstar Omega microplate reader (BMG LABTECH Inc., Cary, NC, USA). A program of 1 min rest followed by 1 min shaking at 700 rpm (double orbital) with fluorescence readings (450/460 nm excitation, 480 nm emission, bottom read, 20 flashes per well, manual gain 1,800) every 15 min for a total of 72 h was carried out for each run. Two thresholds were initially assessed, including 1) the mean value of the first 10 fluorescence readings of the sample well plus 10 SD and 2) the mean value of the first 10 fluorescence readings of the sample well multiplied by 2. The time (h) for a reaction to reach the predefined threshold (ThT), also known as lag time, was recorded and the rate of amyloid formation (RAF) was calculated as the inverse of ThT (1/h). For the ease of data analysis, 0.001 was assigned as the RAF for a negative reaction. All samples were tested at 10^–1^, 10^–2^ and 10^–3^ dilutions under four different experimental conditions including two different types of salt (NaCl and NaI) and with or without ASR1 treatment (NaCl-ASR1 and NaI-ASR1). Three independent test runs were conducted for all platelets samples. A run was considered positive when a sample was tested positive at one or more dilution levels. A platelet sample was considered CWD-positive when it was consistently positive in all 3 runs. Diagnostic sensitivity was defined as the percentage of platelet samples derived from CWD-positive deer that were correctly identified by RT-QuIC. Diagnostic specificity was defined as the percentage of platelet samples collected from CWD-negative deer that were correctly excluded by RT-QuIC.

### Statistical analysis

We estimated the probability of a positive response under four testing conditions using a generalized linear mixed model with a binomial distribution. The response data were the number of positive detections ${y}_{ijk}$ in three trial runs, $n$. This model included a fixed effect for the interaction of salt type $i$ (NaCl or NaI) and ASR1 $j$ (with or without) as well as a random effect to account for variation across individual deer k. Fixed effects for each experimental condition α_ij_ were assigned vaguely, normally distributed priors centered on zero with small precision. Priors for the random deer effect β_k were centered on zero with a uniformly distributed standard deviation between zero and ten.

$${y}_{ijk}\sim binomial\left({p}_{ijk}, n\right)$$

$$logit\left({p}_{ijk}\right)= {\alpha }_{ij} \left[{Salt}_{i}, {ASR1}_{j}\right]+ {\beta }_{k}\left[{DeerID}_{k}\right]$$

$${\alpha }_{ij} \sim normal(0, 0.001)$$

$${\beta }_{k}\sim normal(0,\sigma )$$

$$\sigma \sim uniform(\mathrm{0,10})$$

The probability of a positive test outcome in one run for each combination of Salt and ASR1 condition (${p}_{ij}$) was predicted by taking the inverse logit of the linear portion of the model.

$${p}_{ij}= exp\left({\alpha }_{ij} \left[{Salt}_{i}, {ASR1}_{j}\right]\right)/\left(1+exp\left({\alpha }_{ij} \left[{Salt}_{i}, {ASR1}_{j}\right]\right)\right)$$

The probabilities of at least 1 run, at least 2 runs, and all 3 runs resulting in a positive detection for CWD were derived using the probability of a positive test outcome in a single run and applying it to the following formula:

$$P\left(X=k\right)= {C}_{nk}* {p}^{k}* {\left(1-p\right)}^{n-k}$$

where $n$ is the number of trials or runs, $k$ is the number of positive detections, $p$ is the probability of detection in a single trial for each experimental condition, and ${C}_{nk}$ is the number of ways to obtain $k$ detections in $n$ trials. We applied the formula to estimate the following probabilities:

Probability of no positive detections in 3 trials:

$$P\left(X=0\right)= 1* {p}^{0}* {\left(1-p\right)}^{3-0}$$

Probability of at least 1 positive detection in 3 trials:

$$P\left(X\ge 1\right)= 1-P\left(X=0\right)$$

Probability of at exactly 1 positive detection in 3 trials:

$$P\left(X=1\right)= 3* {p}^{1}* {\left(1-p\right)}^{3-1}$$

Probability of at least 2 positive detections in 3 trials:

$$P\left(X\ge 2\right)= 1-P\left(X=0\right)-P\left(X=1\right)$$

Probability of 3 positive detections in 3 trials:

$$P\left(X=3\right)= 1* {p}^{3}* {\left(1-p\right)}^{3-3}$$

We evaluated the effects of salt type (NaCl or NaI) and ASR1 (with or without) on rate of amyloid formation (RAF) for samples at dilutions of 10^–1^, 10^–2^, and 10^–3^, using a generalized linear mixed model with a log-normal distribution. This included a fixed effect for the three-way interaction between salt type (NaCl or NaI), ASR1 (with or without), and dilution level (10^–1^, 10^–2^, and 10^–3^) and a random effect for deer identity to account for repeated samples from individual deer.

$${log.y}_{ijk}\sim normal\left({mu}_{ijk}, tau\right)$$

$${mu}_{ijkl}= {\beta }_{1ij} \left[{Salt}_{i}, {ASR1}_{j}{Dilution}_{l}\right]+ {\beta }_{2}\left[{DeerID}_{k}\right]$$

We predicted RAF based on estimates of testing condition effects at each dilution level (e.g., NaCl 10^–1^, NaI-ASR1 10^–3^).

$${RAF}_{ijl}= exp\left({\beta }_{1ijl} \left[{Salt}_{i}, {ASR1}_{j}{Dilution}_{l}\right]\right)$$

Models were fit to the data and implemented in a Bayesian framework in R using program JAGS and package jagsUI [34–37]. Convergence was evaluated by examining traceplots and checking for Rhat values of ≤ 1.1 [38].

### Determination of diagnostic sensitivity and specificity

The diagnostic sensitivity was defined as the percentage of true positive samples (samples that were consistently positive in three independent runs) out of all 23 samples collected from CWD-positive deer (ELISA + /IHC +). The diagnostic specificity was determined as the percentage of true negative samples (samples did not show positive responses in all three independent runs) out of all 30 samples collected from CWD-negative deer (ELISA-).

## Results

### RT-QuIC diagnostic sensitivity and specificity

**Fig. 1:**
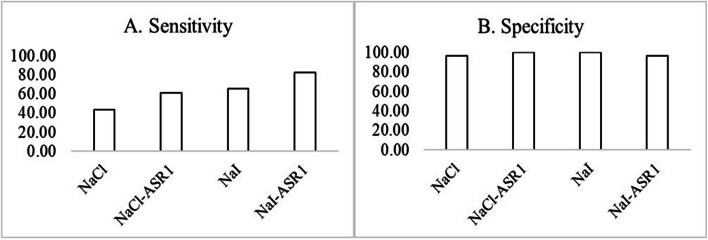
Sensitivity and specificity of RT-QuIC on deer platelet samples. **A** Diagnostic sensitivity defined as the percentage of RT-QuIC + platelet samples derived from CWD + deer. **B** Specificity defined as the percentage of RT-QuIC- platelet samples derived from CWD- deer. Y axis: percent of positive (**A**) or negative (**B**) samples. X axis: testing conditions including NaCl, NaI, NaCl/ASR1, NaI/ASR1. A sample was considered positive when it was tested positive in all three independent RT-QuIC runs at one or more dilutions

Using a stringent threshold (the mean value of the initial 10 readings × 2) and under the following RT-QuIC conditions: NaCl, NaI, NaCl-ASR1, and NaI-ASR1, 10, 15, 14 and 19 of the 23 platelet samples collected from CWD-positive deer were consistently positive while 29, 30, 30, and 29 of the 30 platelet samples collected from CWD-negative deer were RT-QuIC-negative. Thus, the diagnostic sensitivity was 43.48% (NaCl), 65.22% (NaI), 60.87% (NaCl-ASR1) or 82.61% (NaI-ASR1); and the diagnostic specificity was 96.67% (NaCl), 100% (NaI), 100% (NaCl-ASR1), or 96.67% (NaI-ASR1) (Fig. 1A and B). Although higher sensitivities: 69.57% (NaCl), 82.61% (NaI), 69.57% (NaCl-ASR1), and 95.65% (NaI-ASR1) could be achieved using a less stringent threshold (the mean of the initial 10 readings plus 10 × standard deviations), the positive rates for samples collected from CWD-negative deer reached 23.33% (NaCl), 3.33% (NaI), 26.67% (NaCl-ASR1), and 33.33% (NaI-ASR1). To reduced false positive rate, a stringent threshold (the mean of the initial 10 readings × 2) was used to analyze RT-QuIC data throughout this study.

### Rate of positive reactions

The percentage of RT-QuIC positive reactions and the average time to threshold (ThT) for all platelet samples were summarized in Table 1. With samples collected from CWD-positive deer, the percentages of positive reactions were very low at 10^–1^ dilution under all experimental conditions, ranging from 0% to 24.64%. The percentages of positive reactions increased to much higher levels (57.97% to 82.61%) and the time to threshold was shortened (21.55 ± 11.84 to 29.11 ± 19.40) at 10^–2^ dilution, compared to 10^–1^ dilution. At 10^–3^ dilution, the percentages of positive reactions decreased and the time to threshold increased, compared to that of 10^–2^ dilution. Replacing NaCl with NaI and addition of ASR1 pretreatment increased the positive reaction rate at nearly all dilutions. Regarding the 30 samples derived from CWD-negative deer, occasional positive reactions were also seen, especially at 10^–2^ dilution (1.11% to 8.89%) and 10^–3^ dilution (1.11% to 4.44%). However, positive signals were usually detected at late stages of RT-QuIC reactions.

**Table 1:** Percent of positive RT-QuIC reactions and time to threshold (ThT) at different sample dilution levels and under different testing conditions

Dilution	NaCl		NaI		NaCl-ASR1		NaI-ASR1	
	%	ThT	%	ThT	%	ThT	%	ThT
	Samples from CWD + deer							
10^–1^	0.00	(N/A)	4.35	(56.33 ± 4.65)	24.64	(19.75 ± 10.99)	18.84	(37.79 ± 18.19)
10^–2^	57.97	(29.11 ± 19.40)	63.77	(27.64 ± 15.84)	60.87	(22.84 ± 16.27)	82.61	(21.55 ± 11.84)
10^–3^	37.68	(35.10 ± 18.72)	52.17	(28.79 ± 16.98)	34.78	(26.85 ± 15.16)	69.57	(30.19 ± 15.54)
	Samples from CWD- deer							
10^–1^	0.00	(N/A)	1.11	(47.00*)	1.11	(27.50*)	0.00	(N/A)
10^–2^	1.11	(69.25*)	2.22	(56.00 ± 12.37)	4.44	(60.81 ± 7.30)	8.89	(52.03 ± 11.16)
10^–3^	4.44	(56.44 ± 11.15)	1.11	(39.25*)	2.22	(46.25 ± 15.91)	2.22	(46.00 ± 31.11)

### Probability of detecting CWD prion

**Fig. 2:**
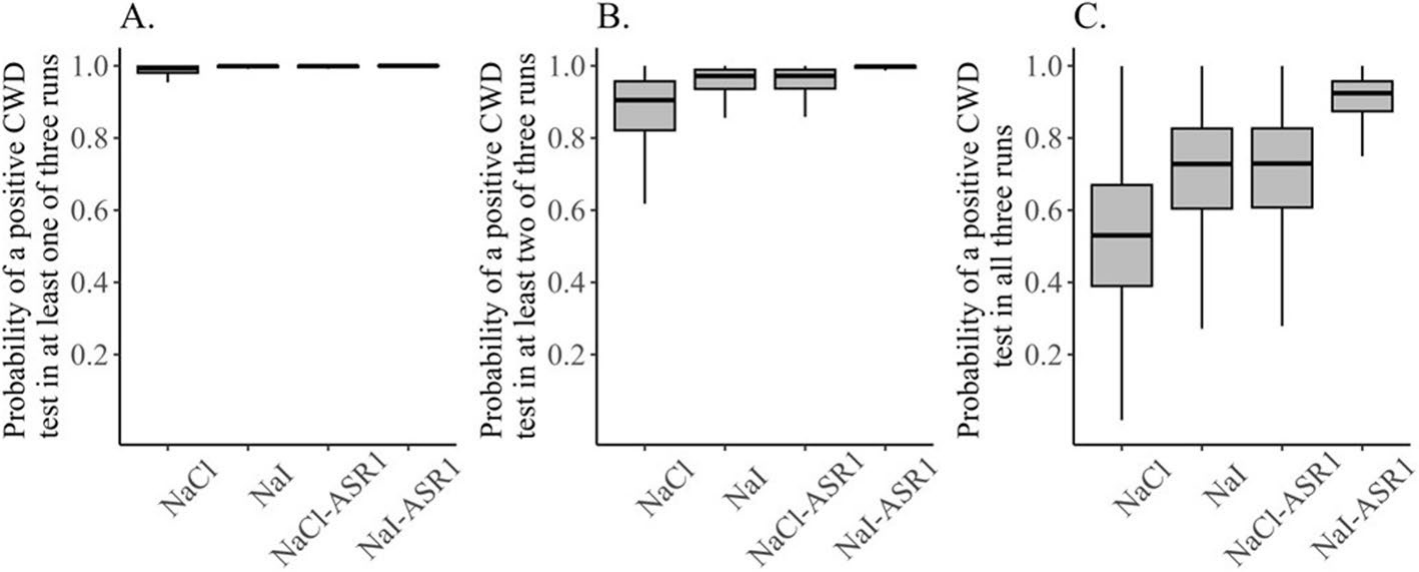
Probability of a RT-QuIC positive detection of CWD prion in at least one, two, and three of three test runs under four experimental conditions: NaCl, NaI, NaCl/ASR1, and NaI/ASR1. Samples were derived from CWD + deer (ELISA + /IHC +)

Statistical analysis of data derived from samples collected from CWD-positive deer indicated that the probability of a positive detection in a single run was greatest under the NaI-ASR1 experimental condition (median = 0.974, 95% CRI: 0.894, 0.996) and lowest under the NaCl condition (median = 0.809, 95% CRI: 0.539, 0.962). Probability of detecting CWD in at least one testing run was nearly perfect under all experimental conditions (Fig. 2A, Table S1). Probability of detecting CWD in at least two testing runs was 0.998 (95% CRI: 0.968, 1.000) under Nal-ASR1 condition and 0.905 (95% CRI: 0.558, 0.996) under NaCl condition (Fig. 2B, Table S1). Probability of detecting CWD in all three test runs for these experimental conditions was 0.924 (95% CRI: 0.714, 0.989) for NaI-ASR1 condition and 0.530 (95% CRI: 0.156, 0.890) for NaCl condition (Fig. 2C, Table S1). There was variation among individual deer in the probability of a positive CWD detection (σ = 2.63, 95% CRI: 1.59, 4.78).

### Rate of amyloid formation

**Fig. 3:**
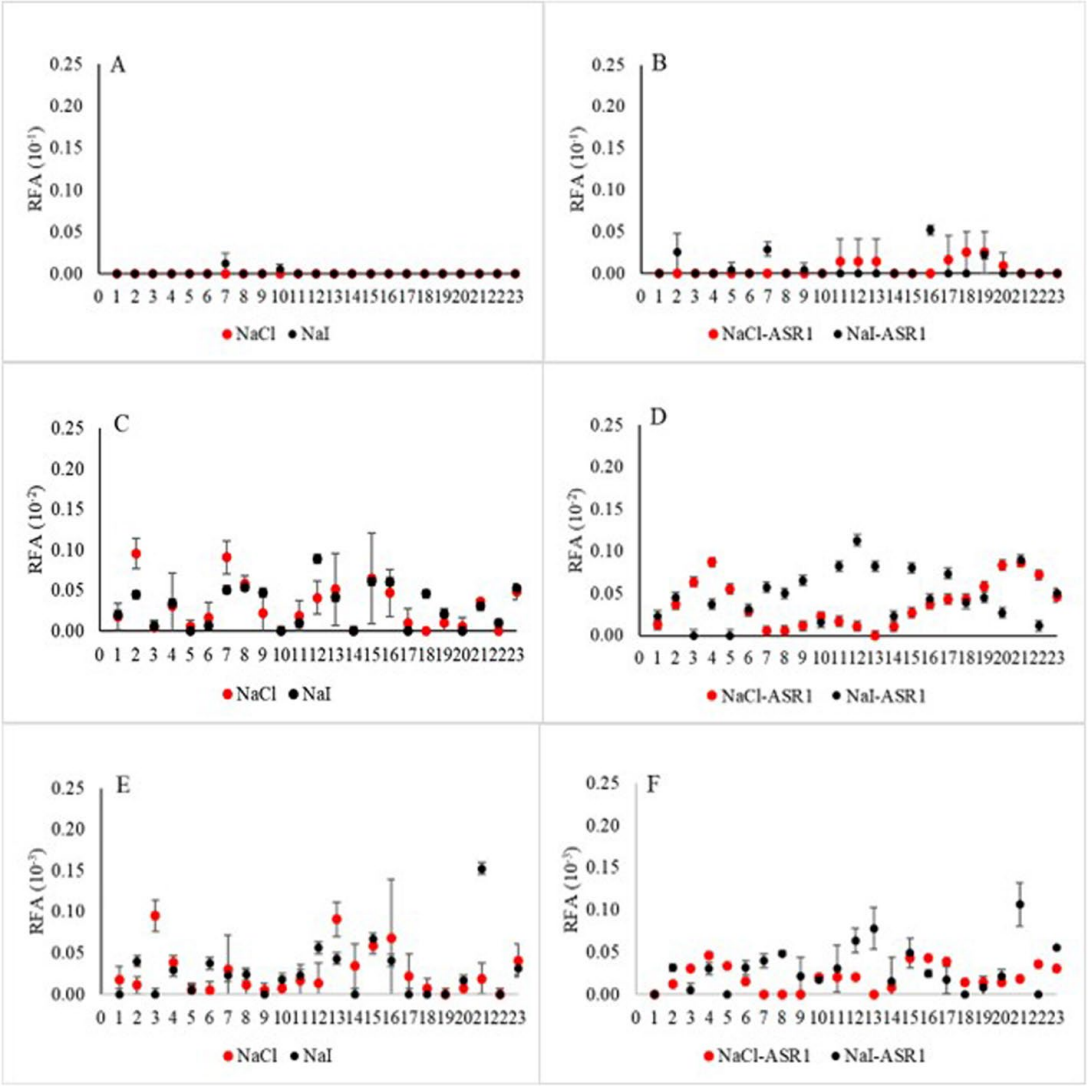
Effect of main ionic component on rate of amyloid formation (RAF) at different sample dilution levels. Serially diluted platelet samples were subjected to RT-QuIC analysis using NaCl- or NaI-based reaction buffer. Y axis: RAF and dilution factor. X axis: samples derived from CWD + deer and tested under different salt conditions

**Fig. 4:**
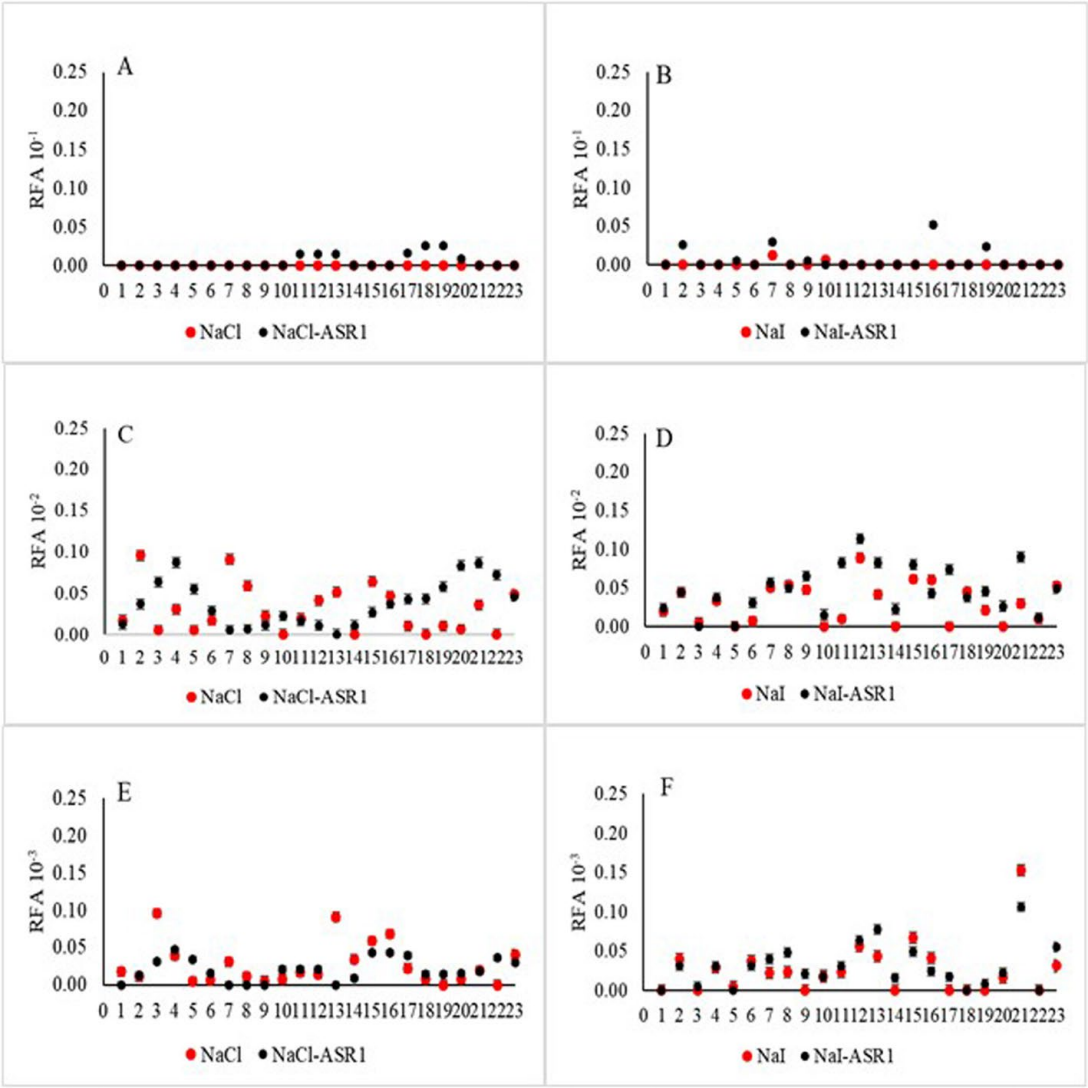
Effect of ASR1 pretreatment on rate of amyloid formation (RAF). Serially diluted platelet samples were subjected to RT-QuIC analysis with or without ASR1 pretreatment. Y axis: RAF and dilution factor. X axis: samples derived from CWD + deer and tested under different conditions

The seeding activity of each platelet sample derived from CWD-positive deer was analyzed at the dilutions and experimental conditions tested in this study. A comparison of RAFs between different experimental conditions indicated that a clear trend of higher RAFs was associated with NaI-ASR1 conditions at 10^–2^ dilution, compared to NaCl-ASR1 and NaCl or NaI without ASR1 Figs. 3 and 4).

**Fig. 5:**
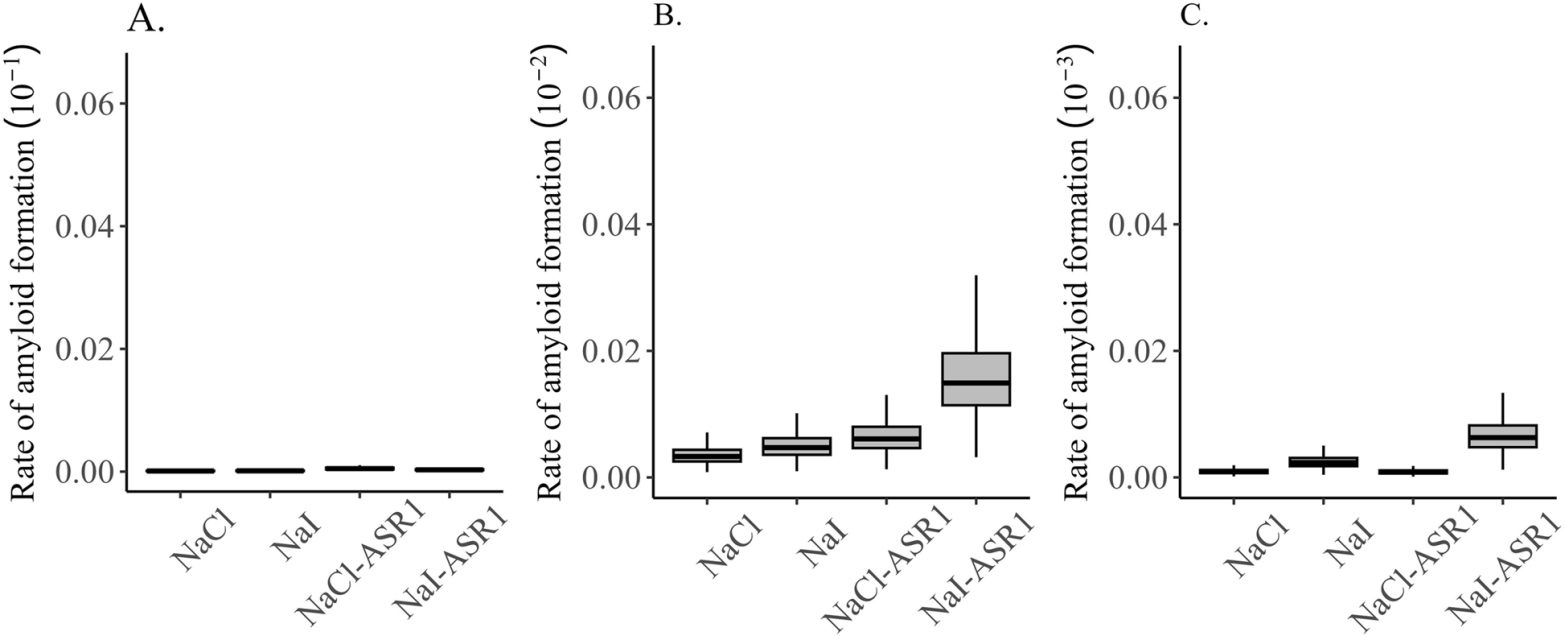
Mean and 95% credible intervals of predicted rates of amyloid formation (RAF) at three dilution levels under four experimental conditions: NaCl, NaI, NaCl/ASR1, and NaI/ASR1. Samples were derived from CWD + deer (ELISA + /IHC +)

Using a generalized linear mixed model with a log-normal distribution indicated that while 95% credible intervals of predicted RAF overlapped, median rate of RAF at 10^–2^ dilution was greatest under the NaI-ASR1 condition (0.01491, 95% CRI: 0.00675, 0.03384) and lowest under NaCl condition (0.00331, 95% CRI: 0.00147, 0.00741, Fig. 5B). Median RAF at 10^–3^ dilution was greatest under NaI-ASR1 condition (0.00629, 95% CRI: 0.00283, 0.01410) and lowest under NaCl-ASR1 condition (0.00084, 95% CRI: 0.00038, 0.00186, Fig. 5C, Table S2). There was variation among individual deer in the probability of a positive CWD detection (σ = 1.43, 95% CRI: 1.04, 2.04).

## Discussion

Although IHC- and ELISA-based detection of PrP^Sc^ in obex or RPLN works well for postmortem CWD diagnosis, it is still challenging to detect CWD prion in antemortem and environmental samples due to several reasons, including difficulty in obtaining suitable samples (e. g. RPLN), low prion concentration in the so called “easily available” samples, and the presence of inhibitors in samples, such as body fluid and feces. Several studies have recently shown the presence of CWD prion in blood samples via the use of prion conversion technologies [6, 18, 19]. Data from one study indicates that CWD blood infectivity is associated with B cells and platelets [6]. Based on the results of these studies, we postulated that platelets can be a suitable type of antemortem diagnostic specimen when the samples are tested using a highly sensitive assay, such as PMCA or RT-QuIC. In this study, we tested platelets, instead of B lymphocytes, because isolation of platelets is relatively simple and does not require any special reagents. However, the buffy coat containing B lymphocytes could also be studied. We focused on ASR1-based preanalytical enrichment which captures all misfolded proteins and removes inhibitors from the sample matrix, and RT-QuIC assay buffer with NaI as the main ionic component. We evaluated the rate of positive reactions, rate of amyloid formation, probability to detect CWD prion in at least one run, two runs and three runs for all 23 samples derived from CWD-positive deer and 30 samples from CWD-negative deer. We used two stringent criteria to determine the status of individual reactions and individual samples. First, we chose the mean value of the initial 10 readings multiplying 2 as the threshold for a reaction to be considered as positive or negative. Second, we counted positivity in all 3 independent runs for a sample to be defined as positive or negative. Based on these standards, the diagnostic sensitivity of RT-QuIC on platelets varied from 43.48% to 82.61% with NaI-ASR1 experimental condition providing the highest sensitivity. The specificity of RT-QuIC on platelets ranged from 96.67% to 100%. Previously, PMCA analysis of blood samples exhibited varying degrees of sensitivity: 53% in RPLN + deer, 96% in obex + /RPLN + deer, and 100% in symptomatic deer that were obex + /RPLN + for CWD [28]. A RT-QuIC study on blood samples derived from TSE-infected and naïve control cervids showed a high sensitivity (> 90%) using a threshold of 5 times SD of the negative control average. In the present study, the samples were from diagnostic submissions without information regarding the stage of disease defined by clinical signs or obex positivity. In addition, setting a threshold based on the readings of negative samples works well for a given research project but does not serve the purpose of routine diagnostics because the limited number of negative controls included in each run do not represent the status of true negative samples. To address variations in sample matrix compositions, we compared thresholds based on the initial readings of individual samples, the mean value of first 10 readings + 10 SD or the mean value of the first 10 readings × 2. Adopting the stringent thresholds reduced overall sensitivity but ensured specificity. It is also important to indicate that postmortem blood samples were collected from hunter-harvested deer at various collection sites and the quality and quantity of the platelets in the samples might not be at an optimal state.

In this study, we observed variations among samples and between testing runs. However, incorporation of ASR1 preanalytical enrichment and NaI as the main ionic source improved the consistency and the percentage of positive reactions, leading to increased diagnostic sensitivity. As shown by the statistical analysis results, the probability of a positive detection in all 3 runs was 0.924 (95% CRI: 0.714, 0.989) under NaI-ASR1 condition and 0.530 (95% CRI: 0.156, 0.890) under NaCl condition. To understand the variations, we investigated the seeding activity at different sample dilution levels and experimental conditions. Overall, RAF was the highest for most samples at 10^–2^ dilution followed by 10^–3^ and then 10^–1^, indicating the presence of inhibitors at 10^–1^. The positive effects of ASR1 and NaI were in line with previous findings that ASR1 selectively captured a diverse range of misfolded proteins, including vCJD prion in human plasma spiked with vCJD brain tissue [26, 27] and NaI enhanced seeding activity of brain and fecal samples [28, 29].

It is noted that our study has several limitations, such as the relatively small number of samples from CWD-positive deer although similar or few samples were tested in the previous RT-QuIC and PMCA studies [18, 19], and the postmortem blood samples being tested. In addition, the incubation time of CWD-positive deer or the stages of disease were unknown which is a common challenge faced by diagnostic laboratories. However, this study focused primarily on the performance of RT-QuIC under various conditions and compared the sensitivity and specificity of RT-QuIC against the initial ELISA/IHC-based diagnostic results. Our data show that blood samples can be used for CWD diagnosis, and the sensitivity may be further improved by testing platelets from fresh blood samples. Based on the outcome of this study, our future research will focus on evaluating the ASR1/NaI RT-QuIC method for the detection of CWD prion in anti-mortem blood samples and fecal samples of captive cervids.

## Conclusion

Incorporation of ASR1 pre-enrichment and NaI as the main ionic component in assay buffer significantly improved CWD RT-QuIC performance consistency, detection probability, and diagnostic sensitivity when postmortem blood samples were tested. The improvements may enable the use of RT-QuIC to detect CWD prion in other fluid samples or environmental samples that usually have a low prion level or contain inhibitors for assay.

### Supplementary Information

**Supplementary Material 1.**

**Supplementary Material 2.**

## Data Availability

No datasets were generated or analysed during the current study.

## Abbreviations

CWD: Chronic wasting disease
RPLN: Retropharyngeal lymph node
PrP^C^: Prion protein-cellular
PrP^Sc^: Prion protein-scrapie
ELISA: Enzyme-linked immunosorbent assay
IHC: Immunohistochemistry
WB: Western blotting
PMCA: Protein misfolding cyclic amplification
RT-QuIC: Real-time quacking induced conversion
rPrP: Recombinant prion protein
ThT: Time to threshold
RAF: Rate of amyloid formation
ASR1: The aggregated specific reagent-1
PBS: Phosphor buffered saline
SDS: Sodium dodecyle sulfate
NaCl: Sodium chloride
NaI: Sodium iodide
