# Supplementary material for: RT-QuIC detection of chronic wasting disease prion in platelet samples of white-tailed deer
Source: BMC Vet Res. 2024 Apr 23;20:152. doi: 10.1186/s12917-024-04005-y (PMC11041042; doi:10.1186/s12917-024-04005-y)
Supplement: Supplementary file 1 — Supplementary Material 1. [file 12917_2024_4005_MOESM1_ESM.docx]

Table S1. Probability of a RT-QuIC positive detection of Chronic Wasting Disease (CWD) in at least one, two, and three of three runs under four experimental conditions, including two salts (NaCl, NaI) and two assays (with or without ASR1).

|  | 1-run |  | 2-runs | 3-runs |
| --- | --- | --- | --- | --- |
| NaCl | 0.993 (0.902, 1.000) |  | 0.905 (0.558, 0.996) | 0.530 (0.156, 0.890) |
| NaI | 0.999 (0.974, 1.000) |  | 0.972 (0.787, 0.999) | 0.728 (0.347, 0.948) |
| NaCl-ASR1 | 0.999 (0.972, 1.000) |  | 0.972 (0.779, 0.999) | 0.729 (0.337, 0.950) |
| NaI-ASR1 | 1.000 (0.999, 1.000) |  | 0.998 (0.968, 1.000) | 0.924 (0.714, 0.989) |
