# Supplementary material for: RT-QuIC detection of chronic wasting disease prion in platelet samples of white-tailed deer
Source: BMC Vet Res. 2024 Apr 23;20:152. doi: 10.1186/s12917-024-04005-y (PMC11041042; doi:10.1186/s12917-024-04005-y)
Supplement: Supplementary file 2 — Supplementary Material 2. [file 12917_2024_4005_MOESM2_ESM.docx]

Table S2. Mean and 95% credible intervals of predicted rates of amyloid formation (RAF) at three dilution levels under four experimental conditions.

| Sample dilution | 10^-1^ | 10^-2^ | 10^-3^ |
| --- | --- | --- | --- |
| NaCl | 0.00010 (0.00005, 0.00023) | 0.00331 (0.00147, 0.00741) | 0.00090 (0.00040, 0.00202) |
| NaI | 0.00013 (0.00006, 0.00029) | 0.00472 (0.00210, 0.01051) | 0.00234 (0.00105, 0.00515) |
| NaCl-ASR1 | 0.00049 (0.00022, 0.00106) | 0.00609 (0.00274, 0.01377) | 0.00084 (0.00038, 0.00186) |
| NaI-ASR1 | 0.00029 (0.00013, 0.00064) | 0.01491 (0.00675, 0.03384) | 0.00629 (0.00283, 0.01410) |
